# PAR2 activation on human tubular epithelial cells engages converging signaling pathways to induce an inflammatory and fibrotic milieu

**DOI:** 10.3389/fphar.2024.1382094

**Published:** 2024-06-28

**Authors:** David A. Vesey, Abishek Iyer, Evan Owen, Danielle Kamato, David W. Johnson, Glenda C. Gobe, David P. Fairlie, David J. Nikolic-Paterson

**Affiliations:** ^1^ Centre for Kidney Disease Research, Translational Research Institute, The University of Queensland at the Princess Alexandra Hospital, Brisbane, QLD, Australia; ^2^ Department of Kidney and Transplant Services, Princess Alexandra Hospital, Brisbane, QLD, Australia; ^3^ Australian Research Council Centre of Excellence in Advanced Molecular Imaging, Institute for Molecular Bioscience, The University of Queensland, Brisbane, QLD, Australia; ^4^ Centre for Inflammation and Disease Research, Institute for Molecular Bioscience, The University of Queensland, Brisbane, QLD, Australia; ^5^ Griffith Institute for Drug Discovery, Griffith University, Nathan, QLD, Australia; ^6^ School of Biomedical Sciences, Faculty of Medicine, The University of Queensland, Brisbane, QLD, Australia; ^7^ Department of Nephrology, Monash Health and Monash University Centre for Inflammatory Diseases, Monash Medical Centre, Clayton, VIC, Australia

**Keywords:** protease, PAR2, TGF-β1, human tubular epithelial cells, transactivation

## Abstract

Key features of chronic kidney disease (CKD) include tubulointerstitial inflammation and fibrosis. Protease activated receptor-2 (PAR2), a G-protein coupled receptor (GPCR) expressed by the kidney proximal tubular cells, induces potent proinflammatory responses in these cells. The hypothesis tested here was that PAR2 signalling can contribute to both inflammation and fibrosis in the kidney by transactivating known disease associated pathways. Using a primary cell culture model of human kidney tubular epithelial cells (HTEC), PAR2 activation induced a concentration dependent, PAR2 antagonist sensitive, secretion of TNF, CSF2, MMP-9, PAI-1 and CTGF. Transcription factors activated by the PAR2 agonist 2F, including NFκB, AP1 and Smad2, were critical for production of these cytokines. A TGF-β receptor-1 (TGF-βRI) kinase inhibitor, SB431542, and an EGFR kinase inhibitor, AG1478, ameliorated 2F induced secretion of TNF, CSF2, MMP-9, and PAI-1. Whilst an EGFR blocking antibody, cetuximab, blocked PAR2 induced EGFR and ERK phosphorylation, a TGF-βRII blocking antibody failed to influence PAR2 induced secretion of PAI-1. Notably simultaneous activation of TGF-βRII (TGF-β1) and PAR2 (2F) synergistically enhanced secretion of TNF (2.2-fold), CSF2 (4.4-fold), MMP-9 (15-fold), and PAI-1 (2.5-fold). In summary PAR2 activates critical inflammatory and fibrotic signalling pathways in human kidney tubular epithelial cells. Biased antagonists of PAR2 should be explored as a potential therapy for CKD.

## Introduction

Chronic kidney disease (CKD) is a growing public health concern that affects between 8% and 16% of the world’s population (Bello et al., 2017). It is associated with excessive healthcare costs and greatly increased risk of cardiovascular disease, kidney failure and death (Al Saleh et al., 2018; Annual Data Report, 2018; Carney, 2020). The incidence of CKD is projected to increase in the years to come as the rates of its major risk factors, diabetes, hypertension, and obesity, continue to rise. Despite the huge social and community burden associated with CKD, treatments are limited and only partially effective (Zou et al., 2021). Developing new effective targeted therapies is therefore a high priority.

All forms of progressive kidney disease, regardless of their etiology, include the key pathological features of inflammation and fibrosis (Lv et al., 2018). These are commonly found together within tubulointerstitial (TI) lesions, and the extent of TI scarring remains the best predictor of progression to kidney failure (Nath, 1992). The proximal tubular epithelial cells are often the first cells to detect insults such as infection, hypoxia, and toxins and subsequently they play a key role in coordinating local host inflammatory immune defenses, and repair mechanisms that allow the reestablishment of tissue homeostasis. It is the excess production of inflammatory cytokines in the setting of ongoing and unresolved kidney injury that exacerbates tissue damage and promotes the transformation of neighboring TI cells, including fibroblasts, pericytes, fibrocytes, macrophages, endothelial cells, into extracellular matrix-protein secreting myofibroblasts that leads to fibrosis (Black et al., 2019).

Serine proteases play important roles in many physiological processes of which digestion and blood coagulation are well known. Their hormone-like actions are less well appreciated and are mediated in part by a unique family of G-protein coupled receptors (GPCR) called protease-activated receptors (PARs). These protease-sensing receptors are widely expressed on the surfaces of cells throughout the body. The four members of this family, PAR1-4, have been associated with epithelial barrier homeostasis, inflammation, pain, fibrosis, and metabolism (Vesey et al., 2007a; Adams et al., 2011). In this study we focus on the second member of this family, PAR2, which is activated by trypsin-like serine proteases including trypsin, matriptase, coagulation-associated proteases, factors VIIa, factor Xa and thrombin (Vesey et al., 2007b; Chung et al., 2013; Vesey et al., 2013). Cleavage of the N-terminal extracellular domain exposes a new N-terminus, SLIGKV in humans, which binds to the body of the receptor and triggers its activation (Barry et al., 2006; Adams et al., 2011). Synthetic peptides of six amino acids which correspond to the new N-terminus, such as 2-furoyl-LIGRLO-NH_2_ (2F), have been widely used as exogenous agonists to investigate roles for PAR2 in physiological and pathophysiological situations (Barry et al., 2007; Barry et al., 2010).

Like with other class A GPCR, PAR2 activation is coupled to intracellular signaling principally by the Gα-proteins, Gq/11, G12/13 and Gi/o at the cytoplasmic/membrane interface. Signaling events include elevations of intracellular Ca^2+^, activation of protein kinase C (PKC) and extracellular signal-regulated kinase (ERK)-1/2 which induce production of inflammatory cytokines (Gq/11), inhibition of adenylyl cyclase activity (Gi/o) and activation of Rho associated kinase, ROCK, which mediates cytoskeletal rearrangements (G12/13) (Adams et al., 2011; Suen et al., 2012; Suen et al., 2014). In addition, signaling independent of G proteins is mediated by the β-arrestin-adaptor proteins which are critical for PAR2 endocytosis and, in some types of cells, the formation of signaling scaffolds. This latter pathway is not evaluated in this investigation although it may well be involved in the regulation of the signaling pathways investigated such as extracellular signal-regulated kinases (Eishingdrelo et al., 2015).

PAR2, also known as F2R like trypsin receptor 1 (F2RL1), is expressed by tubule epithelial cells of the kidney cortex, and enhanced expression by these cells has been reported in various inflammatory and fibrotic diseases (Grandaliano et al., 2003; Palygin et al., 2016; Ma et al., 2019; Bagang et al., 2023). A role in kidney hemodynamic and ion secretion has also been reported (Gui et al., 2003). PAR2 deficiency or PAR2 inhibitors ameliorate diabetic kidney disease, kidney fibrosis, glomerulonephritis, diet induced kidney fibrosis and toxin induced kidney disease in various rodent models (Han et al., 2011; Chung et al., 2013; Hayashi et al., 2017; Wang et al., 2017; Ha et al., 2022; Bagang et al., 2023).

Accumulating evidence points to a role for PAR2 in the pathogenic processes that drive CKD prompting us to explore if PAR2 activation in a primary human tubular epithelial cell culture model induces molecules that promote inflammation and fibrosis.

## Materials and methods

### Materials

The PAR2 antagonist, I-191, and PAR2 peptide agonist, 2f-LIGRLO-NH_2_ (2F), were synthesized and purified by the Institute for Molecular Bioscience, The University of Queensland as previously described (Yau et al., 2016; Jiang et al., 2018). Primary antibodies were from R and D Systems (Minneapolis MN, United States); TGF-β RII (Cat #AF-241-NA), BD Transduction Laboratories (Macquarie Park, NSW, Australia); plasminogen activator inhibitor-1 (PAI-1, Cat #612024), Cell Signaling Technologies (Danvers, MA, United States); phospho-C-Jun (Ser63) XP (#91952) 1/2000, IkB∝ (#9242) 1/2000, phospho-Smad 2 (Ser 245/250/255, Cat 3104) 1/2000, phospho-Smad 2 (Ser465/467, #3108) 1/2000, beta tubulin (Cat # 2128), Santa Cruz Biotechnology; connective tissue growth factor (Cat # sc-365970) 1/2000. Horseradish peroxidase (HRP)-linked secondary antibodies were from R and D Systems (HAF008, HAF017, HAF007) were used at >1/25,000 dilution. ELISA kits used where from R and D Systems (Minneapolis MN, United States); MMP-9 Cat # DY 911, TNF-alpha Cat # DY410, CSF2 Cat # DY215, PAI-1 Cat # DY1786 or BD Transduction Laboratories, human OptEIA kits; TNF Cat # 555212, GMCSF. Recombinant human TGF-β1 (#PHG9204, Gibco), base growth medium, Gibco Dulbecco’s Modified Eagle’s Medium (DMEM)/Ham’s F12 (Cat #11320) and Gibco DMEM (Cat #11995) were from ThermoFisher Scientific (Mt Waverley, VIC, Australia). Pathway specific inhibitors AG1478, SB431542, MLN120B and CC-930 were purchased from Sigma-Aldrich (Merck Life Science Pty Ltd., Bayswater, VIC, Australia).

### Tubule cell isolation and cell culture

Segments of macroscopically and histologically normal kidney cortex (∼10 g) were obtained aseptically from the noncancerous pole of adult human kidneys removed surgically because of small kidney tumors. Patients were otherwise healthy. Informed consent was obtained prior to each operative procedure and the use of human kidney tissue for primary cell culture was approved by the Princess Alexandra Hospital Research Ethics Committee, Brisbane, Australia (ethics number: HREC/12/QPAH/125). The method for isolation and culture of human kidney tubular epithelial cells (HTEC) is described in detail elsewhere (Vesey et al., 2009). Following isolation, cells were cultured in a serum free, hormonally defined DMEM/F12 medium containing epidermal growth factor (20 ng/mL), insulin (5 μg/mL), transferrin (5 μg/mL), hydrocortisone (50nM), triiodothyronine (5pM), selenium (50nM), penicillin (50 U/mL), and streptomycin (50 μg/mL). Cells were routinely cultured in this medium until ready to use. At least 30 donor cell isolates were used in this study.

### Cell treatments

All experiments were performed on confluent passage 1 or 2 HTEC cultured in 48-, 12 or 6-well plates (Corning, NY). Before experimentation, cells were made quiescent by two washes followed by incubation for 24 h in basic media (DMEM medium with antibiotics). On confluence there were approximately two million cells per well of a 6-well plate. Two ml of medium were used per well. There was no significant change in cell number per well with any treatment over the 24 h experimental period. Effects of the PAR2 activating peptide, 2F, on inflammatory/profibrotic cytokine production were assessed by immunoassay and/or Western blotting. Analysis was at 24 h or at times indicated in the figure legends. Specific pathway inhibitors were used to interrogate the signaling pathways involved in PAR2-mediated fibrogenic and inflammatory molecule (TNF, CSF2, MMP9 and PAI-1) induction. The inhibitors were added to cultures 30 min prior to PAR2 activation. EGF receptor kinase AG1478 (0.3 µM), TGF-β receptor I SB431542 (3 µM), JNK inhibitor CC-930 (1.25–10 µM) and inhibitor of nuclear factor kappa B kinase subunit beta, ML120B (1.25–10 µM). The concentrations of these inhibitors used in this study were non-toxic and were those that had previously used in our laboratories or published before by other (Inman et al., 2002; Saifeddine et al., 2015; Yang et al., 2021; Mohamed et al., 2022). The cells and cell conditioned media were harvested according to the various techniques used and, if necessary, stored at −80°C prior to analysis. Harvested medium was centrifuged at 900×g prior to storage. In some cases, the conditioned medium from the cells was concentrated 10-fold using a Nanosep Centrifugal Device with a 3 kDa (OD003C34) molecular weight cut off (Pall, Melbourne, Australia) prior to analysis.

### Quantitative RT-PCR

Cells were grown to confluence and total RNA isolated using a RNeasy Mini Kit, (Qiagen, Hilden, Germany, Cat#: 74104), according to manufacturer’s instructions. cDNA was synthesized using High-Capacity cDNA Reverse Transcription Kit, (Thermofisher Scientific, MA, United States Cat. #: 4368814). A fully validated TaqMan Gene Expression Assay for TNF Hs00174128_m1; CSF2 Hs00929873_m1 and MMP9 Hs00957562_m1, (Thermofisher Scientific, MA, United States), was used with the SensiFAST™ Probe No-ROX Kit (Bioline, London, United Kingdom, Cat# BIO-86020) and LightCycler 480 (Roche Applied Science, Penzberg, Germany) to determine relative gene expression by comparative Ct. The TATA box binding protein, (TBP Hs00427620_m1) was used as an internal control.

### Western blotting

Cells were washed twice with ice-cold phosphate buffered saline (PBS) and lysed with 150 µL of radioimmunoprecipitation assay (RIPA) buffer (Sigma-Aldrich Cat #R0278), containing a protease inhibitor cocktail (Sigma-Aldrich, Cat. #P8340) with phosphatase inhibitor (Thermofisher Scientific Cat # 78442). Cells were further disrupted by sonication, cell debris pelleted by centrifugation (13,000×g, 20 min), and the supernatant collected. The total protein concentrations were measured using the BCA kit (ThermoFisher Scientific, Mt Waverley, Australia). Equal amounts of conditioned medium, (sometimes concentrated), or cell protein (30 µg) were diluted in Bolt LDS sample buffer containing 50 mM dithiothreitol, heated to 55°C (integral membrane proteins) or 70°C (cytosolic proteins) for 10 min, separated on a 4%–12% NuPAGE gel (ThermoFisher Scientific, Mt Waverley, Australia) and electro-transferred to a 0.4 µm polyvinylidene difluoride membrane (Thermo Fisher Scientific, Mt Waverley, Australia). Membranes were blocked with 5% non-fat dried milk in Tris buffered saline (TBS) containing 0.05% Tween (TBST) for 1 h before being incubated overnight with the primary antibody diluted in 5% bovine serum albumin (BSA) in TBST. After washing four times, 5 min each, with wash buffer (TBST), the appropriate secondary HRP-conjugated antibody (dilution >1/25,000, R&D Systems, Minneapolis MN, United States), in blocking buffer was added to the membranes for a 40 min incubation, at room temperature (RT) with gentle agitation. Membranes were washed as above before development with SuperSignal West Pico or SuperSignal WestFemto (ThermoFisher Scientific, Mt Waverley, Australia). A Bio Rad ChemiDoc MP Imaging System was used to capture images. Arcsoft Photo studio 5 was used to create images for this document. The image size, brightness and contrast were adjusted using this software. ImageJ (NIH, United States) was used to estimate band intensity on some images. Pictures are representative images from at least three independent experiments. To control for equal protein loading, membranes were stained with Ponceau red and/or re-probed with a pan-actin monoclonal antibody (1:5,000, Cat No. ACTN05C4, ThermoFisher Scientific, Freemont, CA, United States) or beta-tubulin rabbit monoclonal (1/5,000, Cat No. 2128, Cell Signaling Technology) diluted in 5% BSA in TBST overnight. Following washing, the secondary antibody was used at a dilution of ≥1:25,000. The actin and tubulin bands were visualized as above.

### Statistical analysis

All experiments were performed in at least triplicate from HTEC cultures obtained from at least three separate human donors unless otherwise indicated. Each experiment contained internal controls originating from the same culture preparation. In some cases, for the purposes of analysis, each experimental result was expressed as a change from the control value, which was regarded as 1, and analyzed independently. Results were expressed as means ± standard deviation (SD). Comparisons between two groups were made using paired t-tests. Multiple group comparisons were made by analysis of variance (one-way ANOVA) with Dunnett’s multiple comparison test. GraphPad Prism version 6 was used to construct graphs and for statistical analysis. *p* values ≤0.05 were considered significant. To obtain error bars for western blots the band density for each lane was compared to the internal control lane. The mean fold changes in band density were then calculated. In most case 3 donor experiments were run in parallel at the same time.

## Results

### PAR-2 activation induces secretion of inflammatory and profibrotic cytokines by primary human tubular epithelial cells

**FIGURE 1:**
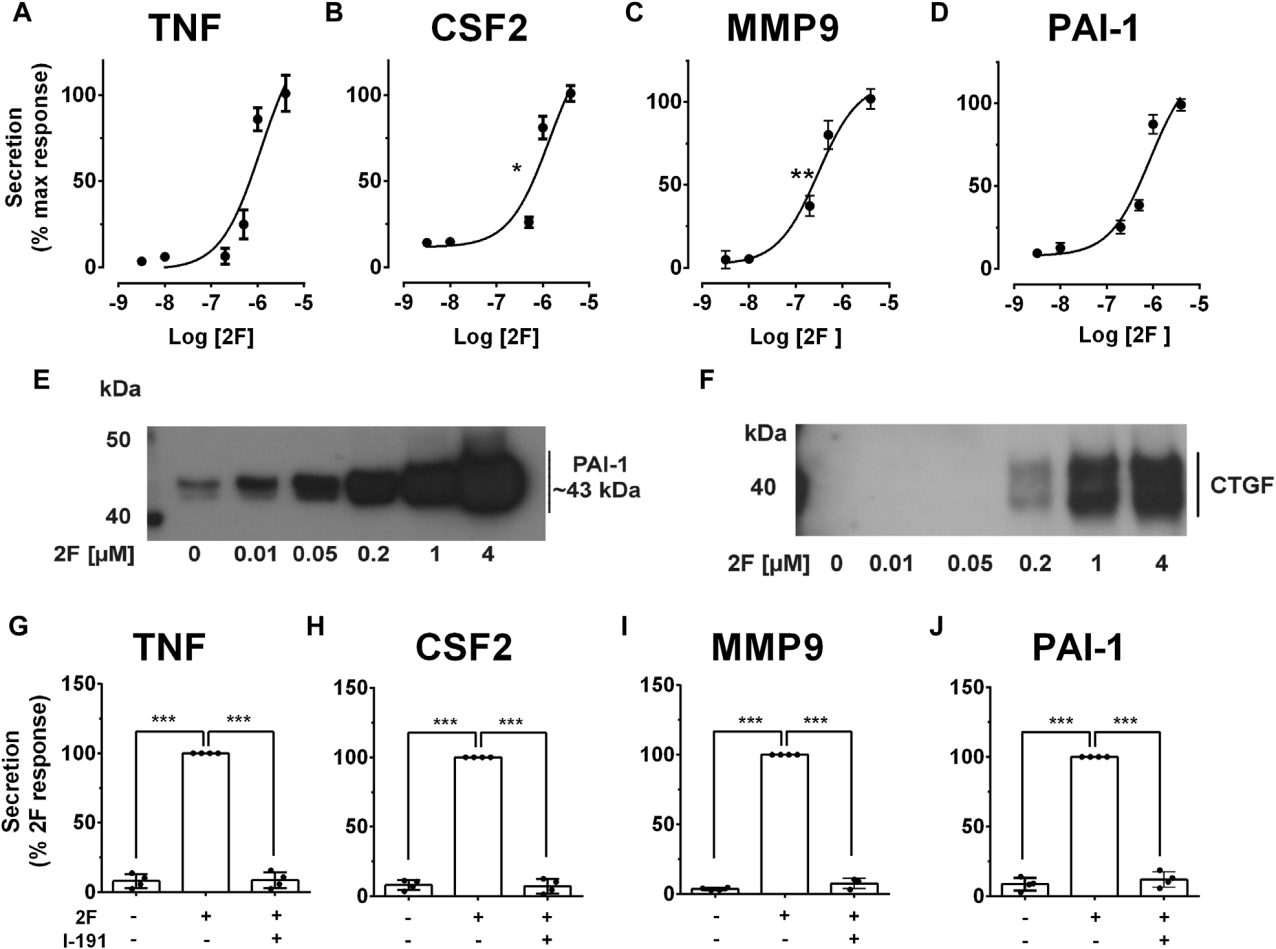
PAR2 activating peptide, 2f-LIGRLO-NH_2_, (2F), induces PAR2 specific secretion of pro-inflammatory and pro-fibrotic factors by HTEC. Cells were grown to confluence in a defined medium and then switched to a basic medium for 24 h. Treatment was with or without 2f-LIGRLO-NH_2_ (2F, 0.01–4 µM) for 24 h. **(A–D)** Concentration dependent secretion of TNF, CSF2, MMP-9 and PAI-1 as measured by specific ELISAs. **(E, F)** Secretion of PAI-1 and CTGF as detected by Western blotting. **(G–J)** PAR2 antagonist I-191 (10 µM) attenuates 2F-induced secretion of TNF, CSF2, MMP-9 and PAI-1 as measured by ELISA. Non-linear regression analysis was used to fit curves in A-D and estimate the IC_50_ for each analyte. Significant increases (*p < 0.05, **p < 0.01, ***p < 0.001) in TNF, CSF2, MMP-9 and PAI-1 were compared to the maximal response **(A–D)** or 2F response **(G–J)** by one-way ANOVA with Dunnett’s multiple comparison test or paired *t*-tests where appropriate. Experiments were repeated three times with cells from different donors. Representative western blots are presented.

Inflammation and fibrosis are common to all forms of progressive kidney disease irrespective of the initiating triggers (Liu, 2011). Whilst they frequently appear together, inflammation often predates fibrosis and is considered to be a major driver of the fibrotic process (Yuan et al., 2022). To examine if PAR2 activation could potentially contribute to these processes, secretion of proinflammatory (TNF, CSF2) and profibrotic molecules (MMP-9, PAI-1, CTGF) by primary cultures of HTEC were measured following PAR2 activation. These molecules have previously been identified to promote kidney inflammation and fibrosis (Eddy, 2009; Al-Lamki and Mayadas, 2015; Becher et al., 2016; Wozniak et al., 2021). A potent synthetic PAR2 activating peptide 2f-LIGRLO-NH_2_ (2F, 0.01–4 µM) induced secretion of TNF, CSF2, MMP9 and PAI-1 in a concentration-dependent fashion with maximal responses between 4-30-fold of control levels at 24 h (Figures 1A–D). At the highest concentration of 2F, the rates of secretion were TNF (∼800 pg/mL/24 h); CSF2 (∼500 pg/mL/24 h); MMP-9 (∼100 pg/mL/24 h); and PAI-1 (∼1 ng/mL/24 h). The calculated EC_50_ for 2F for each molecule in A-D was calculated as 1.2 µM for TNF, 1.4 µM for CSF2, 0.3 µM for MMP9 and 0.8 µM for PAI-1. Secretion of PAI-1 and CTGF into the culture medium in response to 2F treatment are also shown by Western blotting (Figures 1E, F). At lower concentrations of 2F, PAI-1 could be detected as two separated bands of approximately 43 kDa–44 kDa (Figure 1E). At higher 2F concentrations or longer exposures, these bands coalesced to form a broad band between 40 kDa and 50 kDa. Secreted CTGF resolved as two bands of around 40 kDa (Figure 1F). The enhanced secretion of TNF, CSF2 and MMP9 in response to 2F treatment was also shown by quantitative PCR to be preceded by increased synthesis of their respective mRNA transcripts (Supplementary Figure S1). This suggests that there is increased synthesis of these molecules as well as secretion.

A PAR2 antagonist, I-191, that effectively blocks downstream signaling from this receptor was used to confirm that the responses to 2F were specifically mediated by PAR2 activation and not by off target interactions (Jiang et al., 2018). I-191 reduced 2F-induced secretion of TNF, CSF2, MMP-9 and PAI-1 by at least 90% (Figures 1G–J).

### PAR2 activation induces key inflammatory transcription factors NFκB & AP-1 in HTEC

**FIGURE 2:**
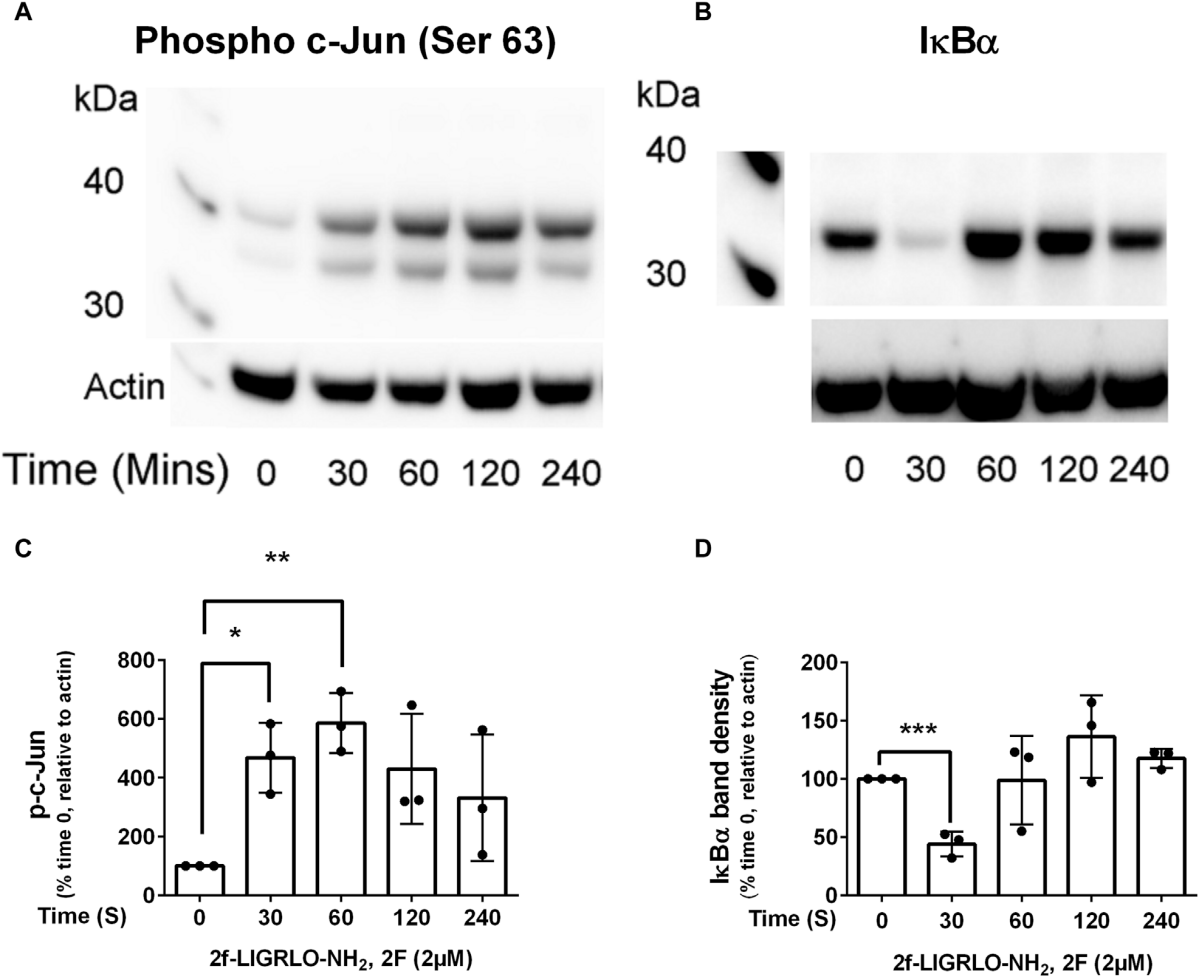
Transcription factors, NFκB and AP-1, are activated by 2f-LIGRLO-NH_2_, (2F). Cells were treated with or without 2F (2 µM) for between 30 and 240 min before harvesting. Twenty µg of cell protein from each treatment was subjected to Western blotting using a phospho-c-Jun **(A)** or IκB∝ **(B)** antibody. Representative blots are shown **(A, B)** and a graphical representation of data generated from the blots **(C, D)**. A paired *t*-test was used to determine the significance of differences between mean band densities. Bars represent the mean ± SD. Significant differences of *p* < 0.05, *p* < 0.01 and *p* < 0.0001 are indicated by *, ** and ***, respectively. *n* = 3.

**FIGURE 3:**
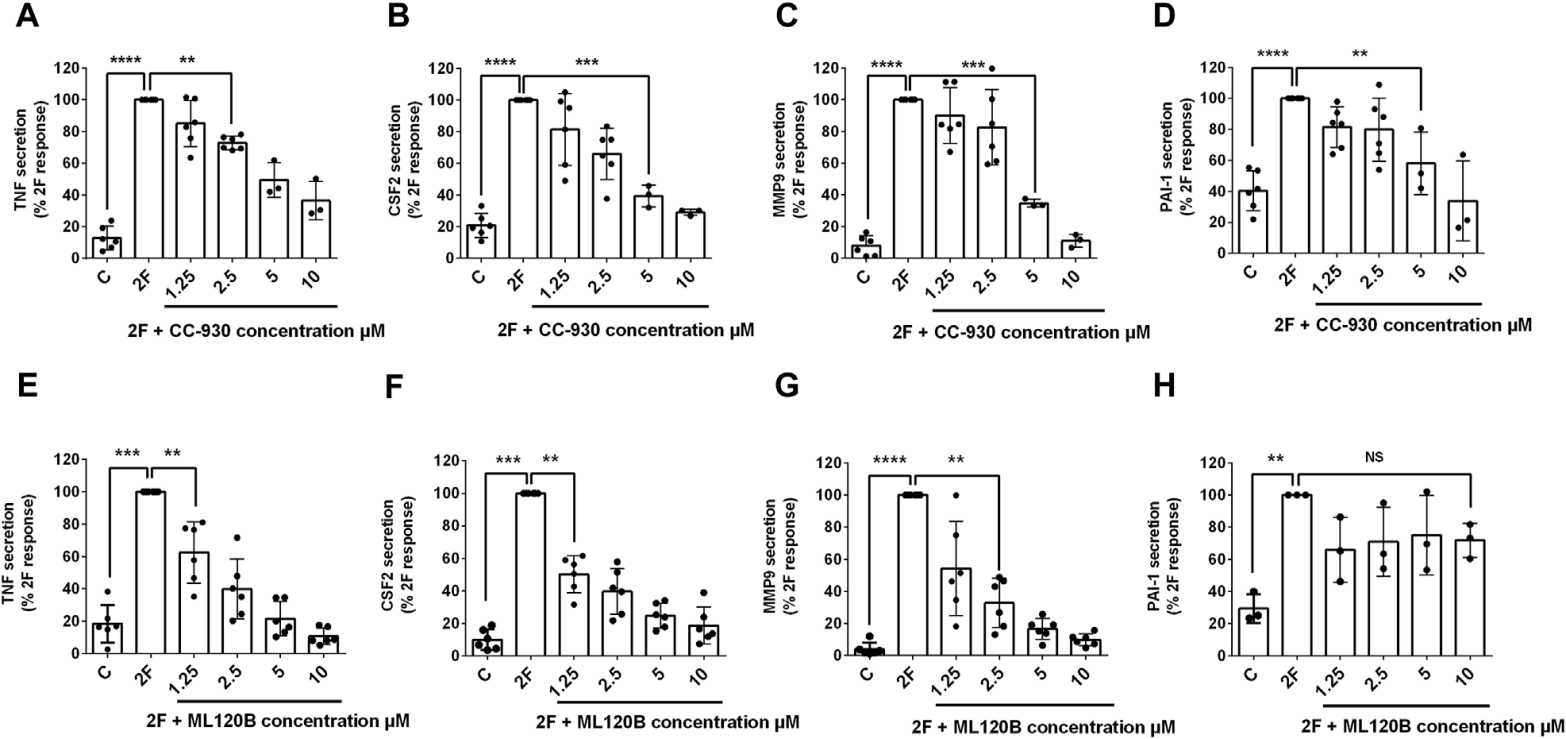
2f-LIGRLO-NH_2_ (2F) induced secretion of TNF, CSF2, MMP-9, and PAI-1 are modulated by antagonists of NFκB and AP-1 activation. Cells were treated with various concentrations of inhibitors CC-930 **(A–D)** or ML120B **(E–H)** for 30 min prior to addition of 2F. Conditioned medium was harvested at 24 h. Secretion of TNF, CSF2, MMP-9 and PAI-1 were measured by specific ELISAs. Data in all bar graphs represent the mean ± SD. Significant (*p < 0.05, **p < 0.01, ***p < 0.001, ****p < 0.0001) increases in TNF, CSF2, MMP-9 and PAI-1 compared to the maximal responses achieved were determined by one-way ANOVA with Dunnett’s multiple comparison test. *n* ≥ 3.

Nuclear factor kappa-light-chain-enhancer of activated B cells (NFκB) and activator protein-1 (AP-1) are transcription factors that are activated in a wide range of inflammatory diseases including those of the kidney (De et al., 2007; Ye et al., 2014; Zhang and Sun, 2015; Liu et al., 2017; Wernig et al., 2017). As they have previously been linked with PAR2 activation, we were interested to determine whether they are activated following PAR2 activation in HTEC and, subsequently, if they were involved in secretion of TNF, CSF2, MMP-9 and PAI. Western blotting was used to determine if the levels of activated nuclear factor of kappa light polypeptide gene enhancer in B-cells inhibitor, alpha (IκB∝) and phosphorylated c-Jun (p-c-Jun) were altered following PAR2 activation. Prior to treatment there was a robust expression of IκB∝ but no or low levels of p-c-Jun (Figures 2A–D). The reverse was the case at 30 min after 2F treatment at which stage IκB∝ was barely detectable, and p-c-Jun was consistently present. The level of p-c-Jun peaked at 60 min and persisted for at least 240 min. IκBα was again present in the cells at 60 min. This suggests that both transcription factors are activated following PAR2 activation in these cells. There was a concentration dependent reduction in 2F induced secretion of TNF, CSF2, MMP-9 and PAI by the JNK inhibitor, CC-930 which blocks phosphorylation of the AP-1 subunit, c-Jun (Figures 3A–D). At 10µM, CC-930 completely inhibited 2F induced secretion of CSF2, MMP-9 and PAI but at this concentration only 70% of TNF secretion was inhibited. In a similar manner MLN120B, which blocks NFκB activation, induced a concentration-dependent (1.25–10 µM), reduction in secretion of TNF, CSF2, and MMP-9 (Figures 3E–G). At 1.25 µM, MLN120B inhibited approximately 57% of 2F induced secretion of TNF, CSF and MMP whereas 98% inhibition was observed at 10 µM MLN120B. Although there appeared to be some reduction in PAI-1 secretion in response to MLN120B, this was not significant at any concentration tested (Figure 3H).

### TGF-β synergistically enhances 2F induced secretion of TNF, CSF2, MMP-9 and PAI-1

**FIGURE 4:**
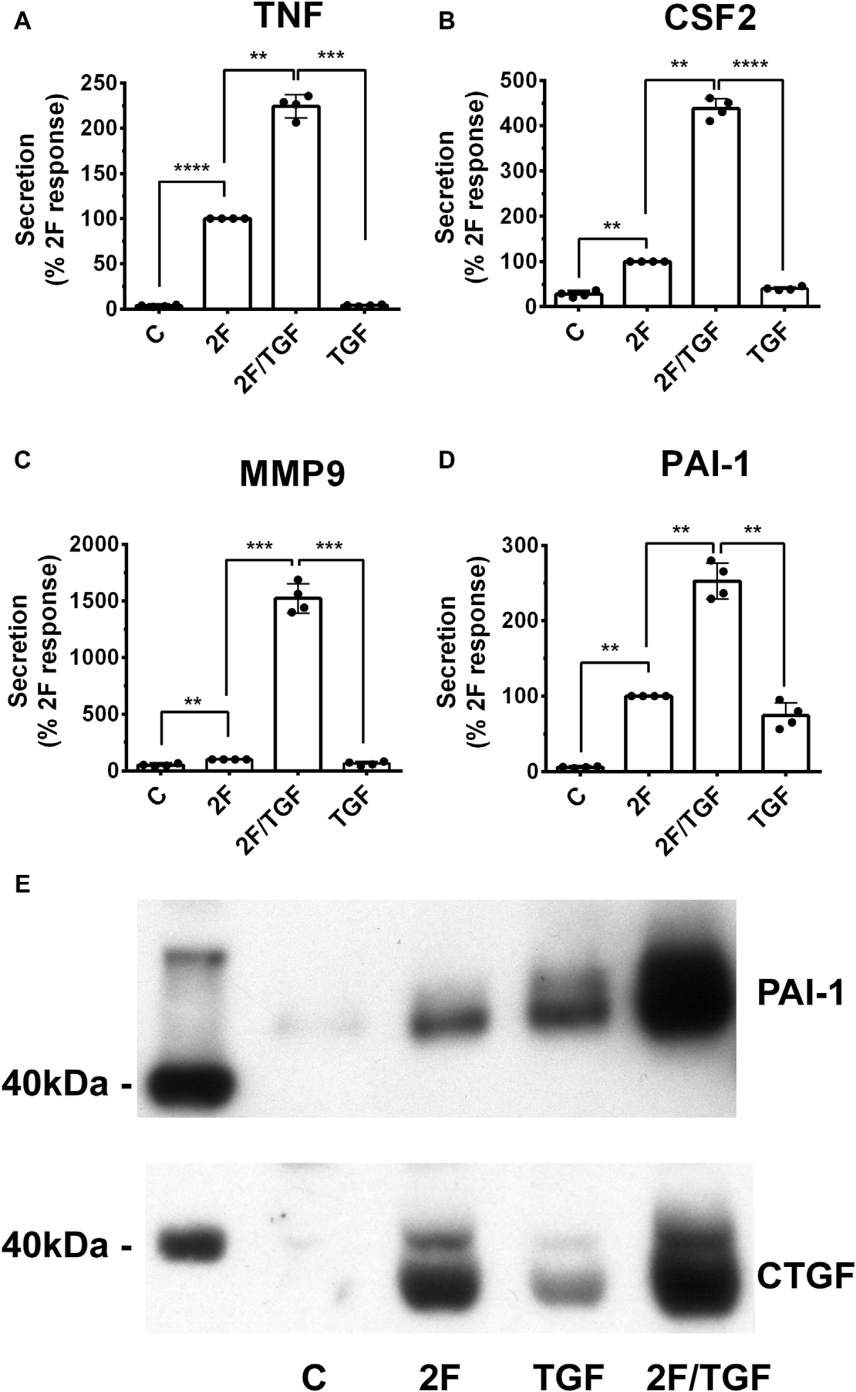
TGF-β1 synergistically enhances secretion of TNF, CSF2, MMP-9, and PAI-1 induced by 2f LIGRLO-NH_2_ (2F) in HTEC. **(A–D)** Cells were treated with 2F (2 μg/mL) with or without TGF-β1 (5 ng/mL) and secretion of TNF, CSF2, MMP-9, and PAI-1 were measured by ELISAs. **(E)** PAI-1 and CTGF secretion as detected by western blotting. All bar graphs represent the mean ± SD. Significant (**p < 0.01, ***p < 0.001, ****p < 0.0001) increases in analytes were compared to the maximal response achieved were determined by one-way ANOVA with Dunnett’s multiple comparison test. *n* = 4.

The role of TGF-β in fibrotic diseases is well documented (Meng et al., 2016; Frangogiannis, 2020). Therefore, we investigated whether TGF-β modulates 2F induced TNF, CSF2, MMP-9 and PAI-1. For this, cells were treated with 2F (2 µM) or TGF-β1 (5 ng/mL) or with both together. Consistent with the above results, stimulation with 2F alone significantly induced secretion of TNF, CSF2, MMP-9 and PAI-1 (Figures 4A–D). When HTEC were treated with TGF-β1 alone there was enhanced secretion of CSF2 (1.4-fold) and PAI-1 (12-fold) compared to control cells; however, TGF-β1 did not significantly induce secretion of MMP-9 or TNF (Figures 4A–D). When TGF-β1 was added together with 2F, secretion of TNF, CSF2, MMP9 and PAI-1 were enhanced 2.2, 4.4, 15 and 2.5-fold respectively, demonstrating a synergistic effect (Figures 4A–D). These results were confirmed by Western blotting for PAI-1 and CTGF (Figure 4E).

### 2F induced phosphorylation of Smad2 at the C-terminal and linker region in a time dependent fashion.

**FIGURE 5:**
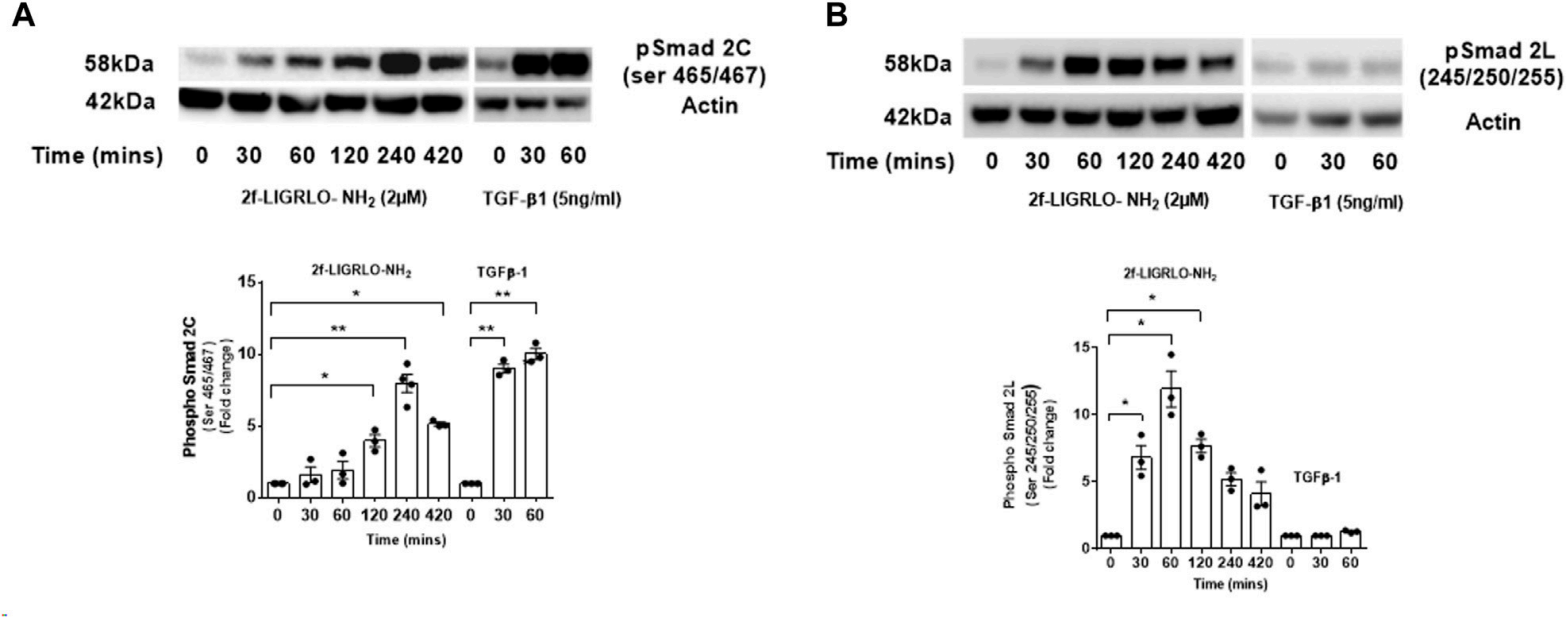
PAR2 activation promotes a time dependent phosphorylation of Smad2 at the C-terminal **(A)** and linker region **(B)** in HTEC. Cells were treated with 2F (2 µM) for up to 420 min or TGF-β1 (5 ng/mL) for 60 min as shown. Total cell lysates (20 µg) were subjected to Western blotting using specific anti‐phospho‐Smad2 (Ser 465/467) and (Ser245/250/255) rabbit monoclonal antibodies and an actin antibody for a loading control. Bar graphs represent band density expressed as fold change from time zero for three independent experiments. Statistical significance was determined using one-way ANOVA with Dunnett’s multiple comparison test. *p < 0.05 or **p < 0.01 compared with untreated cells.

Activation of the TGF-β-Smad signaling pathway is central to the pathogenesis of kidney fibrosis (Meng et al., 2016). The canonical TGF-β signaling pathway is initiated by binding of a TGF-β family member to a TGF-β type II receptor (TGFβRII). A conformational change activates its serine/threonine kinase which then phosphorylates and activates a TGF-β type I receptor (TGFβRI/ALK5). The resulting active heterotetrametric kinase complex can phosphorylate Smad2 and/or 3 which binds to Smad4 and facilitate entry into the nucleus where target gene transcription is induced. By contrast, the non-canonical TGF-β-Smad signaling pathway involves phosphorylation of the linker region of Smad2 and 3 independently of TGFβRI (Meng et al., 2016). Therefore, we examined how PAR2 activation affects Smad2 phosphorylation. Robust Smad2 phosphorylation was observed in both the C-terminal region (serine 465/467) and its linker region (serine 245/250/255) within 30 min of 2F stimulation. Peak levels of phosphorylation were at 60 min for the linker region and 240 min for C-terminal region (Figures 5A, B). Phosphorylation at both sites remained elevated at 420 min. For comparative purposes cells were treated with TGF-β1 for a 60 min period. This demonstrates that the TGF-β/TGFβRII/TGFβR1/Smad signaling pathway is intact. Cells treated with TGF-β1 alone resulted a rapid phosphorylation of Smad2 at the C terminal, but not at the linker region.

### An inhibitor of TGF-β receptor 1/ALK 5, SB431542, but not a TGF-β receptor II blocking antibody, antagonize PAR2-induced TNF, CSF2, MMP-9 and PAI-1

**FIGURE 6:**
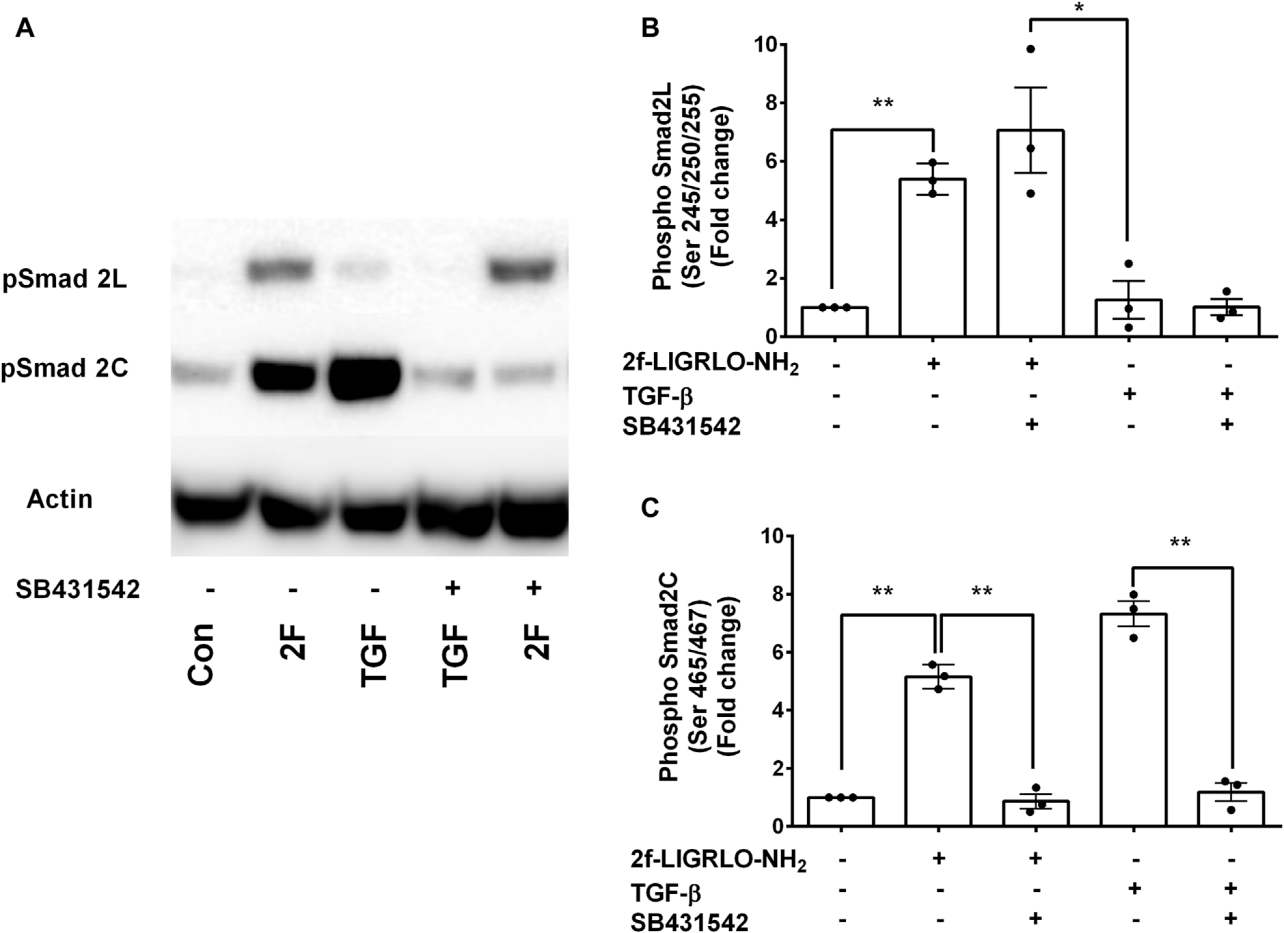
PAR2 induced phosphorylation at the Smad2 C-terminal but not at the Smad2 linker region is blocked by SB431542. Cells were treated with or without the TGF-β receptor I inhibitor SB431542 (3 µM) for 30 min prior to addition of 2F (2 µM) or TGF-β1 (5 ng/ml) for 60 min. **(A)** Total cells lysates (20 µg) were subjected to Western blotting using specific anti‐phospho‐Smad2 (Ser 465/467) and (Ser245/250/255) rabbit monoclonal antibodies and an actin antibody for a loading control. Bar graphs in **(B)** and **(C)** represent band density expressed as fold change from control treatment from three independent experiments. Statistical significance was determined using one-way ANOVA with Dunnett’s multiple comparison test. *p < 0.05 or **p < 0.01 compared with untreated cells.

**FIGURE 7:**
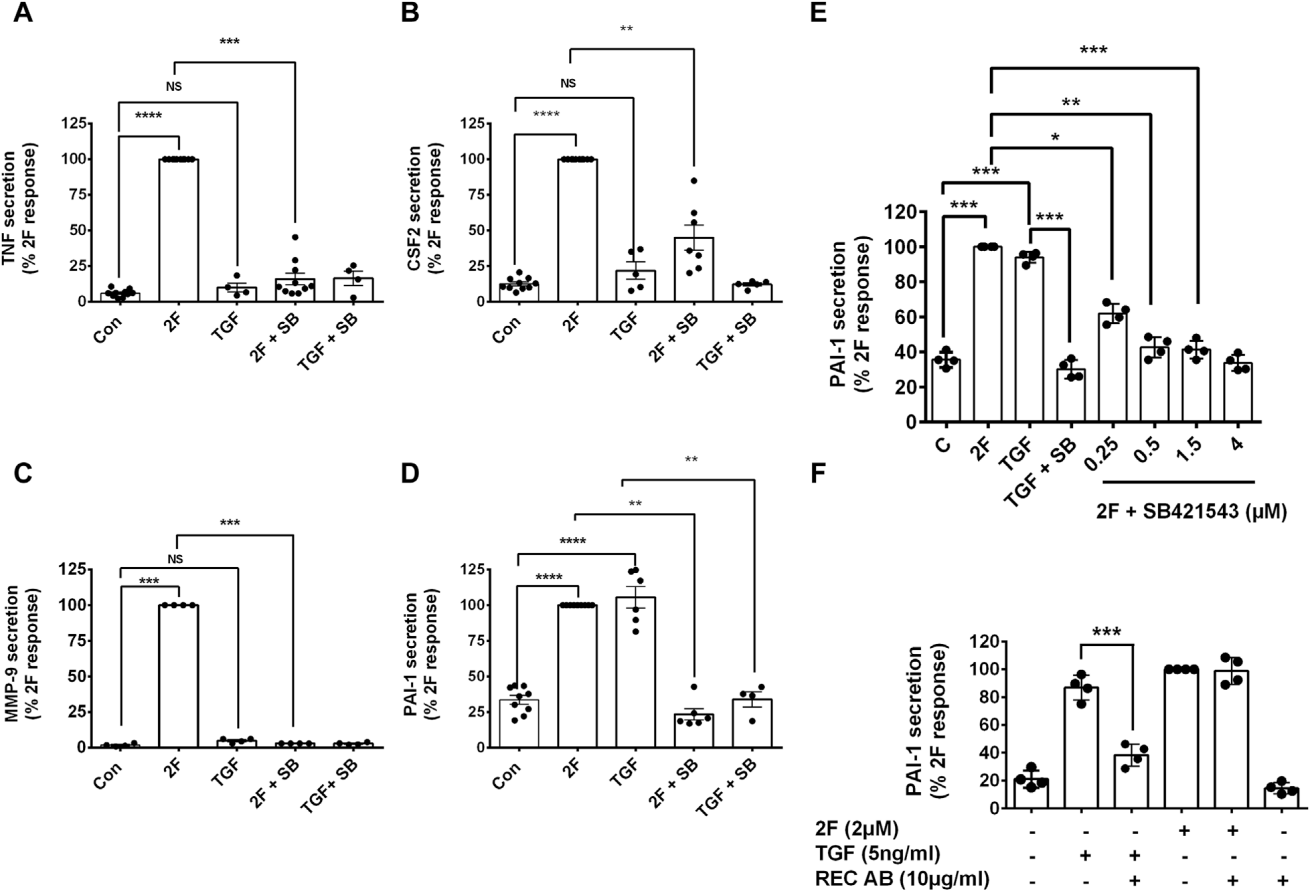
2f-LIGRLO-NH_2_ induced secretion of TNF, CSF2, MMP-9, and PAI-1 is attenuated by inhibition of TGFβRI/ALK 5 by SB431542 but not by a TGFβRII blocking antibody. **(A–E)** Cells were treated with or without inhibitor SB431542 [3 μm **(A–D)**, 0.25–4 μME] for 30 min prior to addition of 2F for 24 h. Secretion of TNF, CSF2, MMP-9, and PAI-1were measured by specific ELISAs. **(F)** Cells were treated with or without a TGFβRII blocking antibody (10 μg/mL) for 30 min prior to addition of 2F or TGF-β1 for 24 h. Secretion of PAI-1 was measured by a specific ELISA. All bar graphs represent the mean ± SD. Quantitative changes in analytes were compared to the maximal response achieved. Significant (*p < 0.05, **p < 0.01, ***p < 0.001, ****p < 0.0001) responses were determined by one-way ANOVA with Dunnett’s multiple comparison test, *n* ≤ 4.

The question of whether PAR2 activation induces secretion of these factors via the canonical or the non-canonical TGF-β signaling pathway was investigated using a TGFβR1/ALK5 inhibitor, SB431542. Treatment of HTEC with SB431542 blocked TGF-β1 induced C-terminal phosphorylation of Smad2, as expected but not the linker region, of Smad2 (Figures 6A–C). Notably, SB431542 treatment dramatically suppressed 2F-induced secretion of TNF, CSF2, MMP-9 and PAI-1 (Figures 7A–D). SB431542 induced a concentration dependent inhibition of 2F-induced PAI-1 secretion (Figure 7E). We next considered whether 2F-induced PAI-1 production involved paracrine secretion of a ligand which activates TGF-βRII. Therefore, a blocking TGF-β receptor type II blocking antibody was added to cultures along with 2F or TGF-β1. Whilst the antibody efficiently blocked TGF-β1 induced PAI-1 secretion, it had no effect on 2F induced PAI-1 secretion (Figure 7F).

### 2F induced secretion of TNF, CSF2, MMP9 and PAI-1 mediated in part be EGF receptor transactivation

**FIGURE 8:**
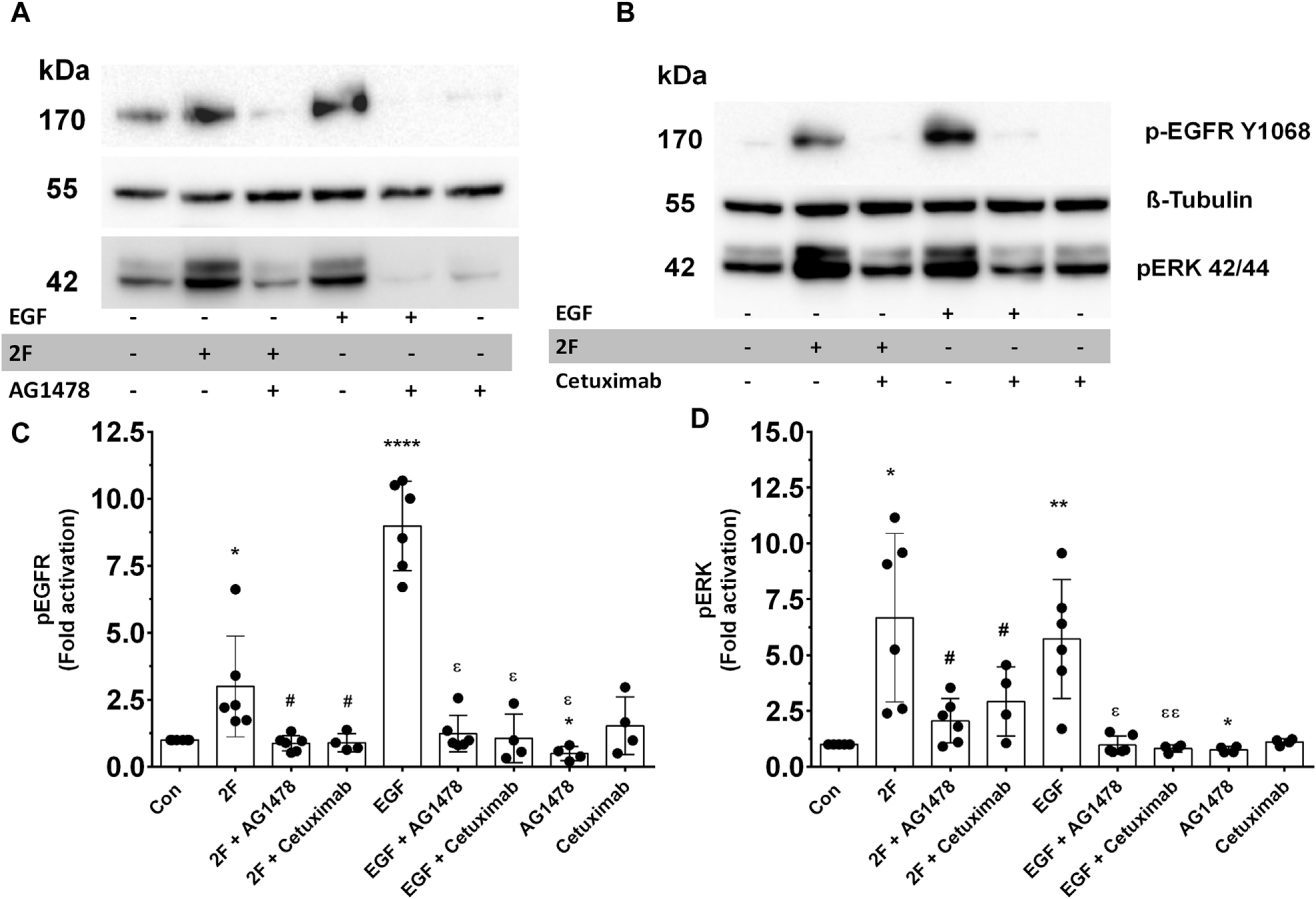
PAR2 activation by 2F induces EGFR and ERK phosphorylation, which is blocked by EGFR tyrosine kinases inhibitor, AG1478, and Cetuximab. **(A, B)** Cells were treated with or without inhibitors AG1478 (0.3 µM) or Cetuximab (50 μg/mL) for 30 min before treatment with 2F (2 µM) or EGF (5 ng/mL) for 10 min. At this time total cell lysates were prepared and processed for Western blotting. Blots were probed with a phospho-EGFR (Y1068), phospho-ERK p42/44 or tubulin antibody (for normalization). Bar graphs in **(C, D)** represent band density expressed as fold change from control treatment for four to six independent experiments Significant changes vs. control (*p < 0.05, **p < 0.01, ****p < 0.0001) or 2F treatment (^#^ *p* < 0.05) or EGF treatment (^**ε**^ *p* < 0.05, ^**εε**^ *p* < 0.01) were determined using one-way ANOVA with Dunnett’s multiple comparison test.

**FIGURE 9:**
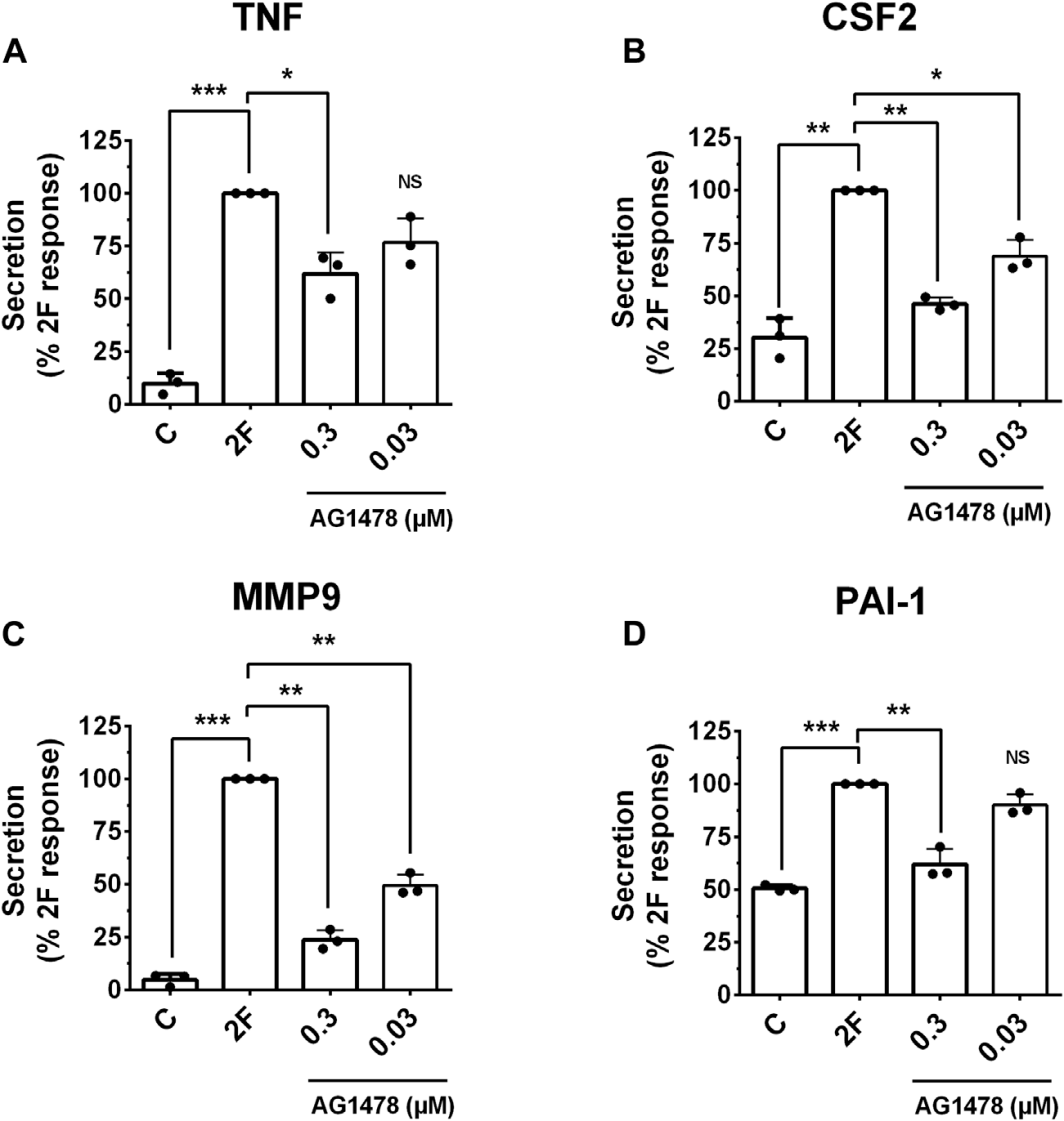
PAR2 induced secretion of TNF, CSF2, MMP-9 and PAI-1 is reduced by the EGFR tyrosine kinase inhibitor, AG1478. Cells were treated with or without the inhibitor AG1478 (0.3 µM or 0.03 µM) for 30 min prior to treatment with 2F (2 µM). Conditioned cell medium was harvested at 24 h and analyzed for TNF **(A)**, CSF2 **(B)**, MMP-9 **(C)** and PAI-1 **(D)** by specific ELISAs. Quantitative changes in analytes were compared to the maximal response achieved (**p < 0.01, ***p < 0.001) using one-way ANOVA with Dunnett’s multiple comparison test. *p < 0.05 or **p < 0.01 compared with untreated cells.

GPCRs are renowned for their ability to transactivate other receptors. This expands the signaling pathways they trigger and produces a wider range of biological responses. PAR2 transactivation of the EGF receptor (EGFR) is widely reported (Wang, 2016; Palanisamy et al., 2021). We thus examined if 2F could induce EGFR phosphorylation in our HTEC cultures and if this could mechanistically be involved in inducing secretion of these factors. 2F induced a rapid and pronounced induction of EGFR phosphorylation on tyrosine (Y) 1068 (Figures 8A–C). In comparison EGF at 5 ng/mL induced a more pronounced phosphorylation of EGFR than 2F. By using an EGFR tyrosine kinase inhibitor AG1478 (0.3 µM) and an anti-EGFR blocking antibody Cetuximab (50 μg/mL) we could block EGFR phosphorylation induced by 2F and EGF. At the concentrations of 2F and EGF used there was a similar robust phosphorylation ERK (Figures 8C, D). Interestingly AG1478 and Cetuximab were more effective at blocking EGF induce ERK phosphorylation than induced by 2F. AG1478, at this non-toxic concentration of 0.3µM, significantly reduced secretion of TNF, CSF2, MMP-9 and PAI-1 by between 40% and 75% (Figures 9A–D).

## Discussion

PAR2 has been touted as a potential therapeutic target for CKD. Support for this has come from experimental models of kidney disease which include a high-fat diet induced CKD (Ha et al., 2022), UUO induced CKD (Chung et al., 2013), adenine-diet induced CKD (Hayashi et al., 2017), immune-mediated glomerulonephritis (Han et al., 2019), glomerulosclerosis (Wang et al., 2017), and diabetic kidney disease (DKD) (Bagang et al., 2023). However, the exact molecular mechanisms by which PAR2 activation drives these different pathologies are not clear. The present investigation demonstrates that PAR2 signalling in human tubular epithelial cells is a potential driver of both inflammation and fibrosis. Using HTEC cultures, we found that activation of PAR2 leads to transactivation and a complex interplay of at least two inter-dependent signaling pathways, EGFR-MAPK and TGFβR1-Smad2, which converge to activate transcription factors, NFκB and AP-1, and trigger expression and secretion of a panel of pro-inflammatory and pro-fibrotic molecules. A PAR2 antagonist, and inhibitors of these pathways or transcription factors, reduce production of these factors. Furthermore, PAR2 activation in combination with TGF-β1 stimulation was found to synergistically enhance secretion of each of these proinflammatory and profibrotic factors.

Primary cultures of HTEC were used in this study as they have previously been shown to express functional PAR2 and respond to both PAR2 activating proteases and peptides (Vesey et al., 2013). These are the most metabolically active cells in the kidney. They possess a high-density of mitochondria, that supply the necessary ATP for reabsorption of approximately two-thirds of the glomerular filtrate (Zhuo and Li, 2013). Their function is also important in kidney disease progression as the extent of TI inflammation and fibrosis is the best predictor of progressive functional decline, even when the predominant disease is glomerular-centric (Nath, 1992). As the first cells of the kidney tubule system to be exposed to the glomerular filtrate, they also have an innate immune sensing function that allows rapid responses to metabolic fluctuations and environmental stresses. This sensing function is mediated by numerous receptors and ion channels and involves altered gene transcription and protein synthesis. Acting in a paracrine or autocrine fashion, secretion of cytokines and growth factors initially instigate protective and reparative processes which can maintained and restore homeostasis. With chronic insults, however, the cells continue to secrete enhanced levels of inflammatory cytokines and chemokines which can trigger expansion of a normally small quiescent population of TI fibroblasts and their transformation into matrix-secreting myofibroblasts (Liu et al., 2018).

In an earlier study we used mRNA expression analysis to investigate the genes induced by PAR2 in HTEC (Vesey et al., 2013). We reported that PAR2 induced a potent pro-inflammatory response with the expression of multiple inflammatory cytokine genes. Here, we show that the peptide 2F induces a concentration-dependent secretion of TNF, CSF2, MMP9, PAI-1 and CTGF by HTEC which was blocked by the selective PAR2 antagonist I-191. Thus, we can conclude that the 2F peptide activates HTEC via selective activation of PAR2.

Secretion of TNF, CSF2, MMP9, CTGF and PAI-1 were measured, as these molecules are known to modulate kidney disease progression in various animal models of CKD. TNF is a potent inflammatory cytokine elevated in the serum and kidney of CKD patients and in experimental kidney disease (Vielhauer and Mayadas, 2007). Blocking its production or antagonizing its interaction with its receptor ameliorates inflammation and fibrosis. CSF2 is a key cytokine known to enhance progression of autoimmune inflammatory diseases such as rheumatoid arthritis and crescentic glomerulonephritis (Lotfi et al., 2019). It has also been reported to be involved in the transition of AKI to CKD (Xu et al., 2019). Expression of MMP9 is enhanced in CKD including AKI and diabetic kidney disease. Circulating levels of active MMP9 are increased in CKD and this correlates with albuminuria (Cheng et al., 2017; Zhao et al., 2017). PAI-1 is a key component of the fibrinolytic system with strong links to kidney inflammation and fibrosis. Circulating levels of PAI-1 are elevated in CKD and deficiency of PAI-1 attenuates interstitial fibrosis and glomerular disease (Flevaris and Vaughan, 2017). Treatment of HTEC with TGF-β1 induces PAI-1 production.

GPCRs are renowned for their ability to transactivate other receptors, particularly those that possess intrinsic tyrosine kinase activity. The GPCR-induced transactivation of the EGF and TGF-β receptors are the most commonly reported (Luo, 2017). Classically, the mechanism for GPCR transactivation of the EGFR involves activation of a metalloproteinase such as ADAM17 which cleaves, and releases cell bound EGFR ligands such as HBEGF, which then activate EGFR (Wang, 2016). Additionally, EGFR can be transactivated by intracellular kinases, including Src, and protein kinase C (PKC). We found that ERK phosphorylation induced by PAR2 and subsequent events leading to secretion of TNF, CSF2, MMP and PAI-1 were to some extent dependent on EGFR transactivation. Both the EGFR kinase inhibitor (AG1478) and cetuximab prevented PAR2 induced EGFR and ERK phosphorylation and AG1478 reduced secretion of TNF, CSF2, MMP and PAI-1. In addition, the TGF-βI kinase inhibitor SB431542 essentially abrogated 2F induced secretion of TNF, CSF2, MMP9 and PAI-1. Therefore, we postulate that PAR2 activation via the 2F peptide induces rapid activation of EGFR and ERK, which then induce TGF-βRI activation and phosphorylation of Smad2; this then leads to activation of the NFκB and AP-1 transcription factors. Of note, a basal level of EGFR and ERK phosphorylation was often found in unstimulated HTEC. We have previously found that a low concentration of the ERK inhibitor, PD98059, significantly reduced the proliferative activity of HTEC (Vesey et al., 2005). This suggests that a base level of EGFR/ERK signalling is necessary to maintain the proliferative and metabolic activity of these cells. It is also worth noting that EGFR activation is not always dependent on ectodomain shedding as membrane bound pro-HB-EGF can activate EGFR by a juxtracrine signaling mechanism (Iwamoto and Mekada, 2000).

TGF-β is upregulated in human and experimental kidney disease and is a central player in development of kidney fibrosis; therefore, we examined if signaling from PAR2 transactivates the TβRI-Smad pathway in our HTEC culture model. This could explain how PAR2 induces PAI-1 and CTGF secretion; both of which are profibrotic and are also induced by TGF-β (Meng et al., 2016).

In this investigation, PAR2 activation induced a time dependent phosphorylation of Smad2 at both the C-terminal (ser465/467) and linker region (ser245/250/255). The C-terminal phosphorylation was dependent on the activity of TGF-βRI, as it was blocked by SB431542. The linker region phosphorylation was not affected by SB431542, which is consistent with the linker region phosphorylation being dependent on other kinases such as the MAPKs (Figure 6). At least in HTEC, 2F-induced phosphorylation of the linker region did not contribute to the secretion of the factors investigated: this is based on the finding that inhibition of Smad2 phosphorylation at the C-terminal, but not at the linker region, was sufficient to block 2F-induced factor secretion. Notably, 2F-induced secretion of these factors was independent of ligand activation of TGF-βRII as shown by the lack of effect of a blocking TGF-βRII antibody. Although enhanced extracellular levels of TGF-β have been reported following PAR2 activation with peptide SLIGKV-NH_2_, active TGF-β was not shown to be released (Vesey et al., 2007b).

A study by Chung et al. (2013), also using human primary kidney tubular cells, reported that PAR2 activation induced transactivation of both the TGF-β and EGF receptors. The transactivation of the TGF-β receptor was indicated by phosphorylation of Smad2/3 and EGFR transactivation by ERK phosphorylation. The latter was inhibited by EGF receptor tyrosine kinase inhibitor, AG1478. Both Smad2 and Smad3 were phosphorylated by PAR2, presumably at the C-terminal, but this was not specified. PAR2 was activated with 2F at 15 µM which is 7.5-fold higher than that used in our studies.

Both NFκB and AP1 are important regulators of inflammation. Previous studies have demonstrated PAR2 induced NFκB activation in a number of other cell types (Macfarlane et al., 2005; Yan et al., 2020). Our results show that activation of these transcription factors is necessary for induction and secretion of TNF, CSF2, MMP9 and PAI-1; and that the EGFR-ERK and TGF-βRI-Smad signaling pathways are involved. Inhibition at different points in this pathway, including EGFR, TGF-βRI, NFκB, and AP-1 attenuates the production of these factors. Crosstalk between the TGF-β signaling pathway and pathways that activate the transcription factors, AP1 and NFkB, has been reported (Luo, 2017). Also, there is significant evidence showing that these transcription factors are regulated by the same intracellular signal transduction cascades and work in a cooperative manner (Oeckinghaus et al., 2011).

Treatment of cells with TGF-β1 itself did not enhance basal MMP9 secretion. On the other hand, 2F alone enhanced MMP9 secretion by 2-fold. When cells were treated with both 2F and TGF MMP9 secretion was enhanced a further 15-fold above 2F-induced secretion. The mechanism of this synergistic secretion is unclear but clearly demonstrates the important role of PAR2 in modifying the known function of TGF-β. Combined treatment of cells with EGF and PAR2 did not synergistically enhance MMP9 secretion in a similar way (data not shown).

Previous studies have demonstrated a synergy between PAR2 and TGF-β1. A report by Ungefroren et al. (2017) conducted using pancreatic cancer cell lines demonstrated crosstalk between these two pathways was necessary for TGF-β induced cell migration. PAR2 expression was found to be necessary but not PAR2-stimulated Gq-calcium signaling. The study by Chung et al. (2013) also highlighted the interaction between PAR2 and TGF-β in kidney cells.

Recent studies have shown that the serine protease inhibitor, camostat mesilate, which is currently in use to treat oral squamous cell carcinoma and chronic pancreatitis, can limit fibrosis in experimental kidney disease by a mechanism which includes attenuating TGF-β expression and collagen IV deposition (Hayata et al., 2012). Two other protease inhibitors, kallistatin and ulinastatin, have also been shown to protect the kidney from development of diabetic kidney disease and acute kidney injury (Yiu et al., 2016; Wei et al., 2021). It is possible that these protease inhibitors also modulate the activity of PAR2 activating proteases. However, it is unclear which proteases are responsible for activation of PAR2 in the kidney *in vivo*. This is despite the identification of more than 25 proteases which activate this receptor *in vitro* (Adams et al., 2011; Heuberger and Schuepbach, 2019).

In summary, PAR2 activation on HTEC induces the production of proinflammatory (TNF, CSF2) and profibrotic factors (MMP9, PAI-1) by transactivation of the EGF and TGF-β receptor signalling pathways. The complex interaction between these pathways leads to activation of the transcription factors NFκB and AP-1 which are critical to this response. These findings support the rationale for further studies to evaluate PAR2 antagonists as drugs to treat CKD.

## Data Availability

The raw data supporting the conclusion of this article will be made available by the authors, without undue reservation.

## Supplementary material

SUPPLEMENTARY FIGURE S1 Activation of PAR2 enhances synthesis of TNF, CSF2 and MMP9 mRNA. Cells were grown to confluence in a defined medium and then switched to basic medium for 24 h. Treatment was with 2F (2 µM) with or without TGF-β1 (5 ng/ml) for 5 h. The levels of TNF **(A)**, CSF2 **(B)**, and MMP-9 **(C)**, mRNA were measured by quantitative PCR. Quantitative changes in mRNA levels relative to were compared to response to control and 2F treatment. Statistically significant changes relative to the control treatment (***p* < 0.01 vs. control ****p* < 0.001 vs. control, # vs. control) and 2F treatment were determined by pair *t*-test.
